# Molecular switches regulating the potency and immune evasiveness of SARS-CoV-2 spike protein

**DOI:** 10.21203/rs.3.rs-736159/v2

**Published:** 2021-10-01

**Authors:** Yushun Wan, Linfen Huang, Xiujuan Zhang, Jian Shang, Stanley Perlman, Lanying Du, Fang Li

**Affiliations:** 1 Department of Veterinary and Biomedical Sciences, University of Minnesota, Saint Paul, MN, USA; 2 Center for Coronavirus Research, University of Minnesota, Saint Paul, MN, USA; 3 Laboratory of Viral Immunology, Lindsley F. Kimball Research Institute, New York Blood Center, New York, NY, USA; 4 Department of Microbiology and Immunology, University of Iowa, Iowa City, IA, USA

**Keywords:** SARS-CoV-2, spike protein, receptor-binding domain, ACE2 binding, viral entry, immune evasiveness, cryo-EM structures

## Abstract

SARS-CoV-2 spike protein plays a key role in viral entry and host immune responses. The conformation of the spike protein can be either open or closed, yet it is unclear how the conformations affect the protein’s functions or what regulate the conformational changes. Using SARS-CoV-1 and bat RaTG13-CoV as comparisons, we identified two molecular switches that regulate the conformations of SARS-CoV-2 spike protein: (i) a furin motif loop turns SARS-CoV-2 spike from a closed conformation to a mixture of open and closed conformations, and (ii) a K417V mutation turns SARS-CoV-2 spike from mixed conformations to an open conformation. We showed that the open conformation favors viral potency by exposing the RBD for receptor binding and viral entry, whereas the closed conformation supports viral immune evasion by hiding the RBD from neutralizing antibodies. Hence SARS-CoV-2 spike has evolved to reach a balance between potency and immune evasiveness, which may contribute to the pandemic spread of SARS-CoV-2. The dynamics between viral potency and invasiveness is likely to further evolve, providing insights into future evolution of SARS-CoV-2.

## Introduction

Coronaviruses have a long history of infecting humans and animals, yet none had caused the same devastation as produced by SARS-CoV-2 (1, 2). For example, a virulent and lethal coronavirus, SARS-CoV-1, yielded a much smaller outbreak in humans in 2002–2003 (3, 4). Numerous human coronaviruses such as NL63-CoV cause common colds annually (5, 6). With an intermediate virulence, SARS-CoV-2 causes a fatality rate that is significantly lower than that of SARS-CoV-1, but much higher than that of NL63-CoV. SARS-CoV-2 carriers show clinical signs that facilitate the spread of the virus: they may develop mild or no symptoms, experience delayed onset of symptoms, develop low levels of neutralizing antibodies, or endure prolonged virus shedding period (7–11). These features contribute to the wide spread of SARS-CoV-2 and severe health outcomes, triggering a global COVID-19 pandemic that is unprecedented in the era of modern medicine. Understanding the molecular determinants of COVID-19 provides important clues to the evolution and cross-species transmission of coronaviruses. A dangerous feature of coronaviruses is their propensity to cross species barriers (12, 13). In fact, coronaviruses similar to human coronaviruses such as SARS-CoV-1 and NL63-CoV have been identified in bats and other animals (14–16). RaTG13-CoV, a coronavirus with ~96% genomic sequence homology with SARS-CoV-2, has been identified in bats (17). Thus, coronaviruses that originate from bats or other animals pose a long-term threat to humans. A comparison of the molecular mechanisms of SARS-CoV-2 and other coronaviruses not only facilitate an understanding of the COVID-19 pandemic, but also shed light on the evolution of coronaviruses, including their cross-species transmission and adaptation to humans.

**Figure 1:**
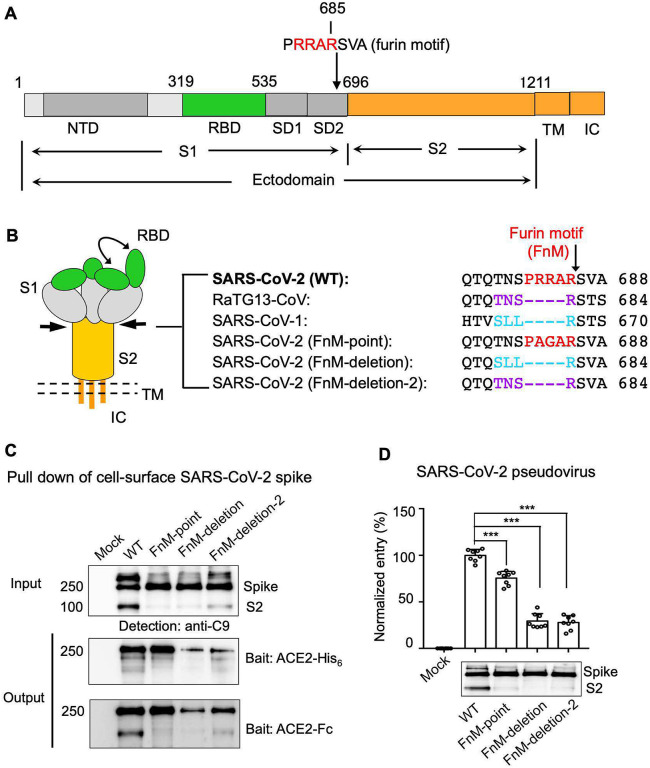
Molecular switch for SARS-CoV-2 spike to close down. (A) One-dimensional schematic representation of SARS-CoV-2 spike. NTD: N-terminal domain. RBD: receptor-binding domain. SD1: subdomain 1. SD2: subdomain 2. TM: transmembrane anchor. IC: intracellular tail. Furin cleavage site is indicated by arrow. (B) Three-dimensional schematic representation of SARS-CoV-2 spike in the pre-fusion structure. The double curve arrow indicates a mixture of open and closed spikes. Double dotted lines represent viral envelope. Arrows indicate location of furin motif (FnM). Also listed is the comparison of the sequences in the furin motif region among SARS-CoV-2, RaTG13-CoV and SARS-CoV-1 spikes. SARS-CoV-2 (FnM-point) spike contains point mutations in FnM. SARS-CoV-2 (FnM-deletion) spike contains FnM deletion as in SARS-CoV-1. SARS-CoV-2 (FnM-deletion-2) spike contains FnM deletion as in RaTG13-CoV. (C) Protein pull-down assay using recombinant human ACE2 as the bait and cell-associated SARS-CoV-2 spike molecules (wild type and mutants) as the targets. Protein levels were detected using Western blot. Top, cell-expressed SARS-CoV-2 spike. Middle, pull-down results using His_6_-tagged ACE2. Bottom, pull-down results using Fc-tagged ACE2. The expression of SARS-CoV-2 spike (which contained a C-terminal c9 tag) was detected using an anti-c9 antibody. Mock, no spike. WT, wild type. (D) SARS-CoV-2 pseudovirus entry into human ACE2-expressing cells. Top, pseudovirus entry efficiency normalized against the expression of the spike (see bottom). Entry efficiency of wild type pseudoviruses was taken as 100%. Bottom, SARS-CoV-2 spike (which contained a C-terminal c9 tag) packaged in pseudoviruses. Its expression was detected by Western blot using an anti-c9 antibody. Individual data points are shown as dots. A comparison (two-tailed Student’s t-test) was performed on data between different groups (n=8). ***P < 0.001. All experiments were repeated independently three times with similar results. Source Data 1: gels/blots

The viral-envelope-anchored spike protein guides coronavirus entry into host cells (18). At the same time, it is a major target for the host immune responses (19). On newly packaged virus particles, the trimeric spike protein has a pre-fusion structure in which three receptor-binding S1 subunits sit on top of a trimeric membrane-fusion S2 stalk (Fig. 1A, 1B). During viral entry, a receptor-binding domain (RBD) in S1 binds to a receptor on host cell surface for viral attachment (20); subsequently S1 dissociates and S2 switches to a post-fusion structure for the fusion of viral and host membranes (18). For the pre- to post-fusion structural change to take place, all coronavirus spikes need to be cleaved by host proteases (21, 22). SARS-CoV-2, SARS-CoV-1, NL63-CoV and RaTG13-CoV can all use ACE2 as the receptor (17, 23–26), but SARS-CoV-2 spike contains two unique features. First, only SARS-CoV-2 spike contains a furin motif at the S1/S2 boundary (27), which allows SARS-CoV-2 spike to be pre-activated by furin from previously infected cells. Second, the pre-fusion structure of SARS-CoV-2 is present in two conformations with approximately equal ratio: an open conformation in which the RBD is exposed and accessible to ACE2 and a closed conformation in which the RBD is buried and inaccessible to ACE2 (28, 29). In contrast, SARS-CoV-1 spike is mainly open and NL63-CoV and RaTG13-CoV spikes are only closed (30–32). Yet despite the extensive structural studies of coronavirus spikes (Table S1), it is unclear what molecular switches regulate their conformations or how the conformational changes affect viral functions and host immune responses.

Here we compared the spike proteins of the three ACE2-recognizing coronaviruses. Using biochemical, pseudovirus, cryo-EM, and animal immunization assays, we first identified the molecular switches that regulate the RBD conformations in SARS-CoV-2 spike. We then demonstrated that whereas the open conformation of SARS-CoV-2 spike increases its potency, the closed conformation allows it to evade host immune responses. Through regulation of its spike’s conformations, SARS-CoV-2 may have struck a balance between viral potency and evasiveness. The dynamics of this balance may further evolve, shedding light on future evolution of SARS-CoV-2.

## Results

To understand the molecular mechanisms that control the spike RBD switching between open and closed conformations, we conducted a comparative study of SARS-CoV-2 and the other ACE2-recognizing coronaviruses. Sequence analysis showed that compared to the closely related SARS-CoV-1 and RaTG13-CoV, only SARS-CoV-2 spike contains a four-residue PRRA insertion ahead of a conserved Arg685 at the S1/S2 junction, constituting the furin motif (FnM) (Fig. 1A, 1B). Hence we introduced mutations to inactivate the FnM in SARS-CoV-2 spike in three ways: (i) point mutations from PRRA to PAGA (i.e., FnM-point); (ii) deletion mutation based on SARS-CoV-1 spike (i.e., FnM-deletion); (iii) deletion mutation based on RaTG13-CoV (i.e., FnM-deletion-2). We then explored whether these FnM mutations affected the conformation and potency of SARS-CoV-2 spike.

To this end, we characterized the capabilities of the FnM mutant spikes of SARS-CoV-2 in binding human ACE2 and mediating viral entry. First, we expressed the wild type and mutant spikes on cell surface (Fig. 1C). The result showed that during the maturation process, a significant amount of wild type spike molecules had been cleaved by furin. In contrast, none of the three types of FnM mutant spikes had undergone significant cleavage, suggesting that inactivation of FnM successfully suppressed furin cleavage of the spikes. Second, we performed a protein pull-down assay using recombinant human ACE2 as the bait and the cell-surface-anchored spikes as the target. For cross validation, both His-tagged ACE2 and Fc-tagged ACE2 were used. We previously showed that this pull-down assay is a reliable method to probe the RBD conformation in cell-surface-anchored spikes, with higher pull-down levels of the spikes associated with more spike molecules in the RBD-open conformation (27). Our results showed that the wild type and FnM-point spikes had similar affinities for ACE2, and both demonstrated much higher affinities for ACE2 than the two FnM-deletion spikes (Fig. 1C). Third, we performed a pseudovirus entry assay where retroviruses pseudotyped with SARS-CoV-2 spike (i.e., SARS-CoV-2 pseudoviruses) were used to enter cells expressing human ACE2 (Fig. 1D). The result showed that the FnM-point spikes mediated pseudovirus entry slightly worse than the wild type spike, suggesting that furin pre-activation had small, albeit significant, impact on SARS-CoV-2 spike’s capability in mediating viral entry. In contrast, both of the FnM-deletion spikes mediated pseudovirus entry much worse than both the wild type spike and FnM-point spike, suggesting that the closed conformation of the spike substantially reduced its capability to mediate viral entry. The data from protein pull-down and pseudovirus entry assays revealed that FnM deletion resulted in decreased potency of SARS-CoV-2 spike, as demonstrated in reduced ACE2 binding and reduced capability of mediating viral entry. These results suggest that due to the FnM deletion, more SARS-CoV-2 spike molecules switched to the closed conformation with reduced potency.

**Figure 2:**
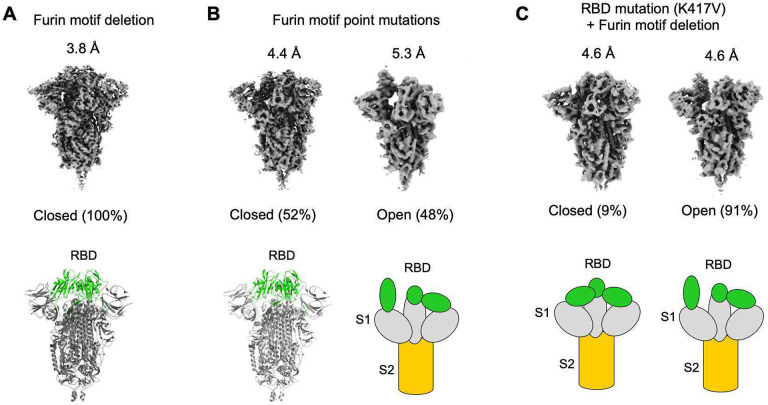
Cryo-EM analyses of the conformations of recombinant SARS-CoV-2 spike ectodomain mutants. These spike mutants contain furin motif deletion (A), furin motif point mutations (B), or both K17V mutation and furin motif deletion (C), respectively. Details of these mutations were explained in Fig. 1B. Their EM density maps, corresponding resolution, and distribution of the particles in open and closed conformations are shown. Atomic models were built for the closed spikes containing furin motif deletion and furin motif point mutations, respectively. Three-dimensional schematic representations are shown for the other spike particles.

Next we directly visualized the conformation of SARS-CoV-2 spike containing the FnM deletion using cryo-EM. To this end, we expressed and purified the ectodomain of SARS-CoV-2 spike containing the FnM deletion (it also contained a C-terminal foldon trimerization tag and two proline mutations in S2, both of which stabilize the pre-fusion structure). As a comparison, we also prepared the ectodomain of SARS-CoV-2 spike containing the FnM point mutation (in addition to the foldon tag and proline mutations). We then collected cryo-EM data on both of these proteins and performed 3-D classifications of the particles based on their conformations (Fig. 2A, 2B; Fig. S1). Our results showed that 48% of FnM-point spike molecules are in the open conformation with one of the three RBDs exposed and the 52% of the molecules were in the closed conformation with all three RBDs hidden (Fig. 2B). This result is consistent with two previous studies showing an approximately equal ratio of open and closed spike molecules (one of the studies involved recombinant FnM-point spike ectodomain and the other virus-surface wild type full-length spike) (28, 29). In contrast, our cryo-EM result showed that all of the FnM-deletion spike molecules were in the closed conformation with all three RBDs hidden (Fig. 2A). Therefore, consistent with our biochemical data, our cryo-EM data confirmed that the FnM deletion caused SARS-CoV-2 spike to switch to the closed conformation.

We further determined the cryo-EM structures of SARS-CoV-2 FnM-deletion spike ectodomain at 3.8 Å and FnM-point spike ectodomain at 4.4 Å (Fig. 2A, 2B; Fig. S2A, Fig. S2B). Overall, the two structures are similar to each other and to the previously determined cryo-EM structures of FnM-point spike ectodomain and virus-surface wild type full-length spike (28, 29). In the trimeric spike structures, each S1 subunit contains an N-terminal domain (NTD), an RBD, and two subdomains (SD1 and SD2); the RBD from one S1 subunit packs against the NTD from another S1 subunit and it also packs against the two RBDs from the other two S1 subunits (Fig. S3A) (28). Moreover, the RBD switches between the open and closed conformations by rotating around a hinge region connecting SD1 and SD2; SD2, which harbors the FnM loop, directly interacts with the hinge region and the NTD (Fig. S3A) (28). Detailed structural analysis revealed that compared to the FnM-point spike, the RBD and NTD in each S1 subunit of the FnM-deletion spike rotated towards each other by ~2.5° (Fig. S4A). Because of this movement, compared to the FnM-point spike, the RBD/NTD interface, the RBD/RBD interface and hence the total interface in trimeric S1 all increased significantly in the FnM-deletion spike, leading to enhanced S1 packing (Fig. S3B). As a comparison, the corresponding interfaces in a previously determined FnM-point spike were similar to those in our FnM-point spike (Fig. S3B) (28). What caused this structural change is not obvious due to the lack of density in the FnM loop and another loop (i.e., anchor loop) in SD2 from all the available structures of SARS-CoV-2 spike. However, the structures of the FnM loop and the anchor loop were resolved in the mouse hepatitis coronavirus (MHV) spike structure that we recently determined (33). Because the MHV and SARS-CoV-2 spikes have overall similar structures (Fig. S4B), we combined the structural information from these two spikes, which revealed an interaction network involving the FnM loop, anchor loop, the hinge region, and the NTD (Fig. S4B). Hence, one possibility is that the FnM deletion disturbed this interaction network and caused the movements of the RBD and NTD, which subsequently led to enhanced S1 packing, reduced dynamics of the RBD and hence the closed spike. Thus, as supported by the biochemical data and 3D classification data, the physical presence of the FnM, instead of furin cleavage per se, leads to open spike molecules by reducing S1 packing.

**Figure 3:**
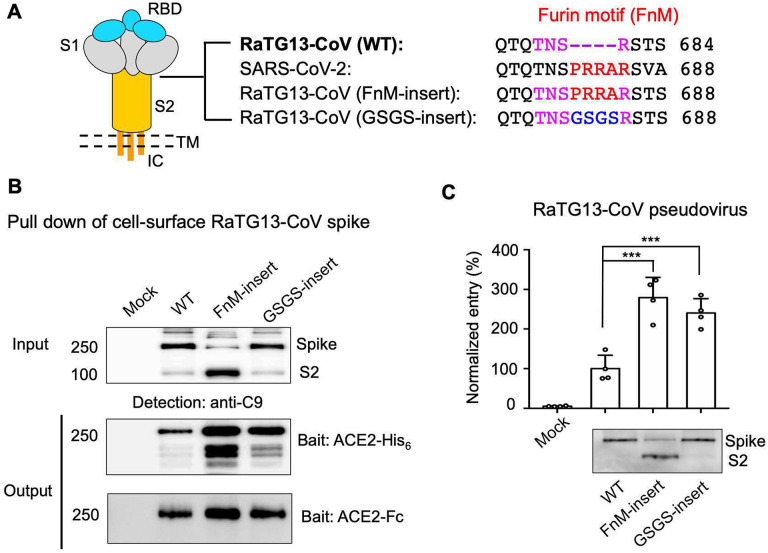
Molecular switch for RaTG13-CoV spike to open up. (A) Three-dimensional schematic representation of RaTG13-CoV spike in the pre-fusion structure with closed RBDs. RaTG13-CoV (FnM-insert) spike contains inserted FnM as in SARS-CoV-2. RaTG13-CoV (GSGS-insert) spike contains an inserted GSGS sequence in the same location as FnM. (B) Protein pull-down assay using recombinant human ACE2 as the bait and cell-associated RaTG13-CoV spike molecules as the targets. Top, cell-expressed RaTG13-CoV spike. Middle, pull-down results using His_6_-tagged ACE2. Bottom, pull-down results using Fc-tagged ACE2. (C) RaTG13-CoV pseudovirus entry into human ACE2-expressing cells. Top, pseudovirus entry efficiency normalized against the expression level of the spike (see bottom). Bottom, RaTG13-CoV spike packaged in pseudoviruses. Data are mean + S.E.M. A comparison (two-tailed Student’s t-test) was performed on data between different groups (n=4). ***P < 0.001. All experiments were repeated independently three times with similar results. Source Data 1: gels/blots

To further understand the relationship between the presence of FnM and the conformation of the spikes, we inserted FnM into RaTG13-CoV spike (i.e., FnM-insert) (Fig. 3A). As a comparison, we also inserted a random sequence, glycine-serine-glycine-serine, into the same location as the inserted FnM in RaTG13-CoV spike (i.e., GSGS-insert) (Fig. 3A). When expressed on cell surfaces, FnM-insert spike, but not wild type spike or GSGS-insert spike, was cleaved by furin (Fig. 3B), confirming the introduction of FnM. We could not obtain recombinant RaTG13 spike ectodomains (wild type or mutants) that were stable enough for cryo-EM analysis (recombinant spike ectodomains are generally less stable than full-length membrane-anchored spikes). Instead, we examined the RBD conformations of the mutant spikes using protein pull-down and pseudovirus entry assays (Fig. 3B, 3C). Compared to the wild type spike, both the FnM-insert and FnM-GSGS RaTG13-CoV spikes bound to human ACE2 with higher affinity and mediated pseudovirus entry more efficiently. Thus, the physical presence of FnM or another random sequence in the FnM loop opens up RaTG13-CoV spike and enhances its potency.

**Figure 4:**
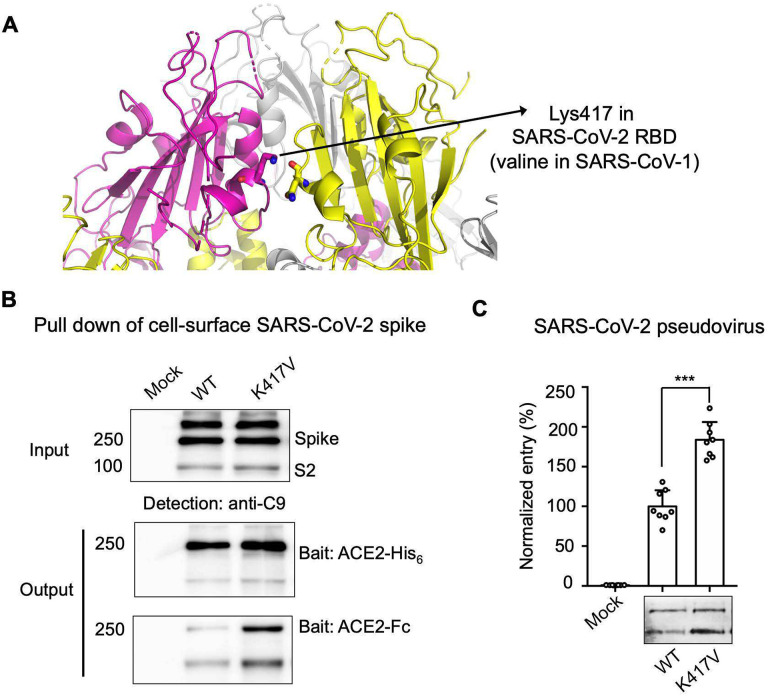
Molecular switch for SARS-CoV-2 spike to open up. (A) Identification of a critical residue Lys417 in SARS-CoV-2 spike that stabilizes the RBD in the closed conformation. The corresponding residue is a valine in SARS-CoV-1. The structure of the closed SARS-CoV-2 spike (PDB 6VXX) is presented from a side view to show three packed RBDs. Each monomeric subunit of the spike trimer is colored differently. (B) Protein pull-down assay using recombinant human ACE2 as the bait and cell-associated SARS-CoV-2 spike molecules as the targets. Top, cell-expressed SARS-CoV-2 spike. Middle, pull-down results using His_6_-tagged ACE2. Bottom, pull-down results using Fc-tagged ACE2. (C) SARS-CoV-2 pseudovirus entry into human ACE2-expressing cells. Top, pseudovirus entry efficiency normalized against the expression level of the spike (see bottom). Bottom, SARS-CoV-2 spike in packaged pseudoviruses. Data are mean + S.E.M. A comparison (two-tailed Student’s t-test) was performed on data between different groups (n=8). ***P < 0.001. All experiments were repeated independently three times with similar results. Source Data 1: gels/blots

Having identified the FnM loop as a key determinant for the conformation of SARS-CoV-2 spike, we asked why SARS-CoV-1 spike is in the open conformation despite its lack of FnM. To address this question, we compared the sequences of SARS-CoV-2 and SARS-CoV-1 spikes in the context of their tertiary structures. We identified residue 417 as potentially a key difference between the two spikes: in the closed SARS-CoV-2 spike, Lys417 in the RBD forms a salt bridge with the RBD from another subunit, stabilizing the RBD in the closed conformation and hence enhancing S1 packing; it becomes a valine in SARS-CoV-1 spike, losing its capability to interact with the other subunit and hence reducing S1 packing (Fig. 4A). We introduced the K417V mutation into SARS-CoV-2 spike, and examined its impact on the conformation of SARS-CoV-2 spike. Both the protein pull-down and pseudovirus entry assays demonstrated that compared to the wild type spike, the K417V mutation allowed more spike molecules to open up for binding ACE2 and mediating viral entry (Fig. 4B, 4C). We could not obtain recombinant SARS-CoV-2 K417V spike ectodomain that was stable enough for cryo-EM analysis. Instead, we prepared recombinant SARS-CoV-2 spike ectodomain containing the K417V mutation and FnM deletion (in addition to proline mutations) (K417V/FnM-deletion). Cryo-EM analysis at 4.6 Å revealed that 91% of the K417V/FnM-deletion spike molecules were open and 9% were closed (Fig. 2C). In comparison, as presented earlier, 100% of the recombinant FnM-deletion spike molecules were closed (Fig. 2A). Therefore, despite lacking FnM, SARS-CoV-1 spike is open due to Val417 and potentially other residues that destabilize the closed conformation of the RBD and reduce S1 packing.

**Figure 5:**
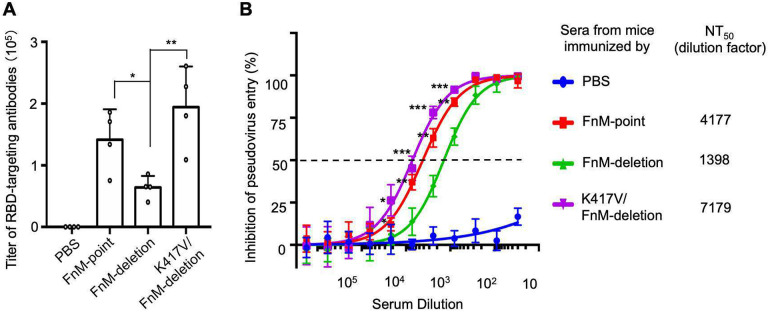
Immune evasion of closed SARS-CoV-2 spike. Mice were immunized with one of the mutant SARS-CoV-2 spikes (4 mice in each group). Subsequently the mouse sera were assayed for titers of RBD-targeting antibodies and neutralizing antibodies. Buffer PBS was used as a negative control in mouse immunization. (A) ELISA for detecting the titers of RBD-targeting IgG antibodies. SARS-CoV-2 RBD (containing a C-terminal His_6_ tag) was coated on ELISA plates, and serially diluted sera from each immunized mouse were added for detection of RBD/IgG binding. The titers were expressed as the endpoint dilutions that remain positively detectable. A titer was determined for sera from each immunized mouse. Data are mean + S.E.M. A comparison (two-tailed Student’s t-test) was performed on sera between the FnM-deletion group and one of the other experimental mouse groups (n=4). **P < 0.01. *P < 0.05. (B) Pseudovirus entry inhibition assay for detecting the titers of neutralizing antibodies. SARS-CoV-2 pseudoviruses were used to enter human ACE2-expressing cells in the presence of serially diluted sera from each group of immunized mice (sera from mice within each immunization group were pooled together for this assay). NT_50_ of sera was determined as the dilution factor that led to 50% inhibition of pseudovirus entry. High NT_50_ suggests high titers of neutralizing antibodies in the sera. Data are mean + S.E.M. A comparison (two-tailed Student’s t-test) was performed on sera at individual dilution point between the FnM-deletion group and one of the other experimental mouse groups (n=3). ***P < 0.001. **P < 0.01. *P < 0.05. All experiments were repeated independently three times with similar results.

To understand how the RBD conformations of SARS-CoV-2 spike affect host immune responses targeting the RBD, we immunized mice with one of the following three recombinant SARS-CoV-2 spike ectodomains: FnM-deletion spike, FnM-point spike, and K417V/FnM-deletion spike (in addition to the proline mutations in all of them). Four weeks after the initial immunization, the mice were further boosted with the same immunogen. Ten days after the second immunization, mouse sera were collected. We measured the amounts of RBD-specific antibodies in the mouse sera using ELISA. The result showed that K417V/FnM-deletion spike and FnM-point spike induced significantly more RBD-specific antibodies than FnM-deletion spike (Fig. 5A). We further measured the amounts of neutralizing antibodies in the mouse sera using pseudovirus entry inhibition assay. The result showed that K417V/FnM-deletion spike and FnM-point spike induced significantly more neutralizing antibodies than FnM-deletion spike (Fig. 5B). These data confirm that more molecules of K417V/FnM-deletion spike and of FnM-point spike are in the open conformations than FnM-deletion spike. They also reveal that compared to open spikes, closed spikes trigger lower levels of RBD-targeting antibodies and neutralizing antibodies and hence their RBDs and spikes are more evasive to the host immune system.

To summarize, we investigated the molecular switches regulating the conformation of SARS-CoV-2 spike protein. We used four different experimental approaches: pull-down of cell-surface spikes, cryo-EM analysis of recombinant spike ectodomains, spike-mediated pseudovirus entry, and immunization of mice with recombinant spike ectodomains. To date, several other studies also investigated the conformations of SARS-CoV-2 spike using cryo-EM (28, 29, 31, 34, 35), some of which gave different ratios of open and closed spikes probably due to differences in sample preparations and/or protein constructions. The ratio of open and closed spikes in our FnM-point construct is similar to two other studies: a study that examined the full-length virus-anchored SARS-CoV-2 spike (which is likely the most physiologically relevant) (29) and a study that used the same protein construct and similar protein preparation to the current study (28). Importantly, our cryo-EM analysis is consistent with our three other experimental approaches. These different experimental approaches complement each other and make this study among the most comprehensive in investigating the conformations of SARS-CoV-2 spike.

## Discussion

Several molecular features of SARS-CoV-2 may have contributed to the COVID-19 pandemic. Among them are features involving SARS-CoV-2 spike: high ACE2-binding affinity of the RBD, the presence of the furin motif at the S1/S2 boundary, and the RBD switching between open and closed conformations (26–28). The molecular mechanisms for the first two have been well established in previous research on SARS-CoV-1 and MERS-CoV (MERS-CoV spike contains a furin motif) (18, 20, 36). These two features facilitated SARS-CoV-1 and MERS-CoV, respectively, to infect humans. In this study we examined the molecular mechanisms that regulate the conformations of SARS-CoV-2 spike. Our study showed that the conformations of coronavirus spike proteins are regulated through one or a few molecular and structural switches. Our study also clarified the functions of these conformational changes of coronavirus spikes. It has implications for the structure, function and evolution of coronavirus spikes and for the current and potentially future coronavirus infections.

How do the conformations of SARS-CoV-2 spike impact viral entry and host immune responses? Among the coronavirus spikes whose tertiary structures are available, only three showed a significant presence of open conformations in cryo-EM studies: those from SARS-CoV-2, SARS-CoV-1, and MERS-CoV (Table S1). All three are novel coronaviruses that recently infected humans. In contrast, coronaviruses with closed spikes all have established infections in their respective hosts (the spikes would need to open briefly for receptor binding). This difference suggests that the open spike may facilitate novel coronaviruses to infect humans. Consistent with this hypothesis, here we showed that compared to closed spikes, open spikes mediate more efficient receptor binding and viral entry. On the other hand, SARS-CoV-2 spike has a balanced ratio between open and closed conformations, which may enhance immune evasion during its transmission in humans. Consistent with this hypothesis, here we showed that more spike molecules in the closed conformation correspond to decreased inductions of RBD-targeting antibodies and neutralizing antibodies in mice. Together, these findings demonstrate that the presence of open and closed conformations of its spike allows SARS-CoV-2 to balance its potency and immune evasiveness.

How has SARS-CoV-2 spike evolved to reach the balance of potency and evasiveness? Through comparative studies of the spikes from SARS-CoV-2, SARS-CoV-1 and RaTG13-CoV, we identified two molecular switches for the open and closed conformational changes of SARS-CoV-2 spike: the physical presence of the furin motif loop and the mutation of residue 417, both of which regulate S1 packing. Thus, one or several structural changes in coronavirus spikes can function as molecular switches for the conformations of coronavirus spikes. Other molecular determinants may also exist to control the opening and closing of coronavirus spikes, but these two naturally occurring molecular determinants help understand the evolution of coronavirus spikes. Coronaviruses that have evolved molecular switches to open up their spike may have an advantage in spreading efficiently in the infected host species. In contrast, those that have evolved mechanisms to close down their spikes may have an advantage in establishing evasive and long-lasting infections in the infected host species.

These results further our understanding of the molecular mechanisms for the COVID-19 pandemic. First, adaptation of SARS-CoV-2 RBD to human ACE2 and furin cleavage of SARS-CoV-2 spike both play important roles in the transmission of SARS-CoV-2 in humans. Second, a balanced open and closed RBD conformations of SARS-CoV-2 spike enable the virus to be both potent and immune evasive. Unlike the stepwise accumulation of point mutations in the RBD for enhanced ACE2 binding, the molecular switches for the RBD conformations of the spike allow more drastic and efficient control of ACE2 binding and viral entry. The opening up of its spike likely facilitates SARS-CoV-2 to gain infection potency and spread efficiently in humans. Moreover, with balanced conformations of its spike, SARS-CoV-2 is also immune evasive. This feature of SARS-CoV-2 may contribute to the relatively long incubation time, harder-to-detect symptoms (even asymptomatic infections), low neutralizing antibodies, or long virus shedding period in patients; these clinical symptoms of patients may further contribute to the wide spread of SARS-CoV-2. Therefore, the balanced potency and immune evasiveness of SARS-CoV-2 spike may contribute to the wide spread of SARS-CoV-2.

Our findings also provide insights into how SARS-CoV-2 may further evolve. When SARS-CoV-2 first entered humans, its spread met little immune resistance. The more open and potent spike gave the virus an advantage in spreading widely. Several months in the pandemic, a D614G mutation allowed more SARS-CoV-2 spike molecules to open up (37, 38), a sign that the virus was gaining more potency (39). Eventually, however, as infection cases rise and vaccinations get underway, SARS-CoV-2 may evolve towards better immune evasiveness. This may happen through an increase in the proportion of closed spikes, making the virus more immune evasive but less potent. If that happens, SARS-CoV-2 may become an endemic (but milder) virus like NL63-CoV (NL63-CoV RBD binds to human ACE2 with high affinity, but is hidden in the closed spike) (25, 30, 40). This study showed that just one or a few structural changes in the spike protein can significantly impact the dynamics between viral potency and evasiveness. This makes coronaviruses a current and future danger to human health. Understanding the molecular determinants that regulate the potency and evasiveness of coronaviruses is critical not only for our understanding the current COVID-19 pandemic, but also for monitoring and preparing for potential future coronavirus pandemics.

## Materials and Methods

### Plasmids

All of the protein constructs in this study were cloned into pcDNA 3.1 vector (Life Technologies). SARS-CoV-2 spike (GenBank accession number QHD43416.1), SARS-CoV-1 spike (GenBank accession number YP_009825051.1), RaTG13-CoV spike (GenBank accession number QHR63300.2), and human ACE2 (GenBank accession number NM_021804) were all synthesized (GenScript Biotech) and cloned into the vector containing a C-terminal c9 tag. SARS-CoV-2 spike ectodomains (residues 1–1211) were cloned into the vector containing mutations of interest, in addition to two proline mutations in S2 (K986P, V987P), a C-terminal foldon trimerization tag, and a C-terminal His_6_-tag. SARS-CoV-2 spike RBD (residues 319–535) and SARS-CoV-1 spike RBD (residues 306–521) were cloned into the vector containing an N-terminal tPA signal peptide. Human ACE2 ectodomain (residues 1–615) were cloned into the vector containing either a C-terminal His_6_-tag or Fc-tag.

### Protein expression and purification

All of the recombinant proteins were expressed in 293F cells (Thermo Fisher) using a FreeStyle 293 mammalian cell expression system (Life Technologies) as previously described (41). In brief, the His-tagged proteins were collected from cell culture medium, purified using a Ni-NTA column (Cytiva Healthcare), purified further using a Superdex gel filtration column (Cytiva Healthcare), and stored in a buffer containing 20 mM Tris pH 7.4 and 200 mM NaCl. The Fc-tagged protein was purified in the same way as the His-tagged proteins, except that the protein A column replaced the Ni-NTA column in the procedure.

### Pseudovirus entry

Pseudoviruses were packaged as previously described (42). Briefly, pcDNA3.1(+) plasmid encoding one of the full-length coronavirus spike genes (wild type or mutant) was co-transfected into HEK293T cells with helper plasmid psPAX2 and reporter plasmid plenti-CMV-luc at a molar ratio of 1:1:1 using Lipofectamine 3000 (Life Technologies). The produced pseudoviruses were harvested 72 hours post transfection and then were used to enter HEK293T cells expressing human ACE2. After incubation at 37°C for 5 hours, medium was replaced and cells were incubated for an additional 48 hours. Cells were then washed with PBS and lysed. Aliquots of cell lysates were transferred to Optiplate-96 (PerkinElmer), followed by addition of luciferase substrate. Relative light unites (RLUs) were measured using EnSpire plate reader (PerkinElmer). In the meanwhile, the amounts of pseudovirus-packaged spikes were measured by western blot using an anti-c9 antibody and then were quantified using Fiji (https://imagej.net/). The RLUs were then normalized against the amounts of pseudovirus-packaged spikes. All of the measurements were carried out in quadruplicates.

For pseudovirus entry inhibition, mouse sera were serially diluted in DMEM media and then mixed with SARS-CoV-2 pseudoviruses. Subsequently the mixtures were added to HEK293T cells expressing human ACE2 for the pseudovirus entry assay. The fitted curves and the 50% neutralizing antibody titers (NT_50_) were calculated using the Graphpad Prism program. All the measurements were carried out in triplicates.

### Western blot

Pseudoviruses were mixed with SDS loading buffer and then were incubated at 95°C for 10 min. Samples were run in a 10% SDS Tris-Glycine Gel and transferred to a PVDF membrane. An anti-c9 or anti-His_6_ monoclonal primary antibody (1:1000 dilution, Santa Cruz Biotech) and a horseradish peroxidase-conjugated mouse secondary antibody (1:10,000 dilution, Jackson Laboratory) were used for Western blotting. A LAS-4000 imager was used to develop images.

### Protein pull-down assay

Protein pull-down assay was performed using a Dynabeads immunoprecipitation kit (Invitrogen) as previously described (41). Briefly, 10 μL of Dynabeads, either for His_6_-tagged proteins or for Fc-tagged proteins, were washed with PBS buffer and then were incubated with either 8 μg ACE2-His (human ACE2 with a C-terminal His_6_ tag) or 10 μg ACE2-Fc (human ACE2 with a C-terminal Fc tag), respectively. Subsequently, ACE2-bound beads were washed with PBS buffer plus 0.05% Tween-20 (PBST) and then were aliquoted into different tubes for later use. To prepare cell-associated coronavirus spike, HEK293T cells were transfected with pcDNA3.1(+) plasmid encoding coronavirus spike (containing a C-terminal c9 tag). 48 h after transfection, the spike-expressing cells were lysed in immunoprecipitation assay buffer using a sonicator and then centrifuged. The supernatants containing solubilized coronavirus spike (or purified recombinant coronavirus RBDs for RBD pull-down assay) were incubated with the ACE2-bound beads (spike or RBD was in excess of ACE2). Then beads were washed with PBST buffer, and the bound proteins were eluted using elution buffer. The samples were then subjected to Western blot analysis and detected using an anti-C9 tag antibody or anti-His_6_ tag antibody.

### Cryo-electron microscopy (cryo-EM)

For sample preparation, aliquots of recombinant SARS-CoV-2 spike ectodomain (3 μl; 0.35 mg/ml; in buffer containing 10 mM Tris pH7.4 and 100 mM NaCl) were applied to glow-discharged CF-2/1–4C C-flat grids (Protochips). The grids were then plunge-frozen in liquid ethane using a Vitrobot system (FEI Company).

For data collection, images were recorded using a Gatan K2 Summit direct electron detector in super resolution mode, attached to a FEI Titan-Krios TEM. The automated software SerialEM was used to collect movies at 22,500x magnification and at a defocus range between 0.6 and 2.6 μm. 1847 movies were collected for FnM-point spike ectodomain, 4784 movies were collected for FnM-deletion spike ectodomain and 4563 movies were collected for K417V/FnM-deletion spike ectodomain. Each movie had an exposure of 7.822 e-/Å2/s fractionated in 40 frames of 8 second exposure. Data collection statistics are summarized in Table S2.

For data processing, whole frames in each movie were corrected for motion and dose compensation using MotionCor2 (43). The final images were bin-averaged to reach a pixel size of 1.04 Å. The parameters of the microscope contrast transfer function were estimated for each micrograph using GCTF (44). Particles were automatically picked and extracted using Gautomatch (http://www.mrc-lmb.cam.ac.uk/kzhang/Gautomatch/) and RELION (45) with a box size of 300 pixels. For FnM-deletion spike ectodomain, 728,804 particles were initially extracted and subjected to 2D alignment and clustering using RELION. The best classes were then selected for an additional 2D alignment. ~5,000 best particles were selected for creating the initial 3D model using RELION. 107,268 particles selected from 2D alignment were then subjected to 3D classification. The best class with 65,302 particles was subjected to 3D refinement to generate the final density map with C3 symmetry. For FnM-point spike ectodomain, 583,127 particles were initially extracted and subjected to 2D alignment and clustering using RELION. The best classes were then selected for an additional 2D alignment. ~5000 best particles were selected for creating the initial 3D model using RELION. 52,134 particles selected from 2D alignment were then subjected to 3D classification for open and closed conformations. The best open-conformation class with 21,894 particles and the best closed-conformation class with 23,849 particles were subjected to 3D refinement to generate the final density maps with C1 symmetry and C3 symmetry, respectively. For K417V/FnM-deletion spike ectodomain, 1,267,763 particles were initially extracted and subjected to 2D alignment and clustering using RELION. The best classes were then selected for an additional 2D alignment. 124,721 best particles were selected for creating the initial 3D model using RELION. 26,126 particles selected from 2D alignment were then subjected to 3D classification for open and closed conformations. The best open-conformation class with 101,413 particles and the best closed-conformation class with 9,502 particles were subjected to 3D refinement to generate the final density maps with C1 symmetry and C3 symmetry, respectively. The final density maps were sharpened with modulation transfer function of K2 operated at 300keV using RELION. Reported resolutions were based on the gold standard Fourier shell correlation (FSC) = 0.143 criterion. Fourier shell correction curves were corrected for the effects of soft masking by high-resolution noise substitution (46).

### Model building and refinement

The initial model of the SARS-CoV-2 spike ectodomain was obtained by fitting the cryo-EM structure of a previously determined SARS-CoV-2 FnM-point spike ectodomain (PDB ID: 6VXX) into our cryo-EM density maps using UCSF Chimera and Coot (47, 48). Manual model rebuilding was performed using Coot based on the well-defined continuous density of the main chain. Side chain assignments were guided through the density of bulky amino acid residues. The structural model of SARS-CoV-2 spike ectodomain was refined using Phenix (49) with geometry restrains and three-fold noncrystallographic symmetry constraints. Refinement and model rebuilding were carried out iteratively until no further improvements were achieved in geometry parameters and model-map correlation coefficient. The quality of the final model was analyzed using MolProbity (50). The validation statistics of the structural models are summarized in Table S2.

### Calculation of interface area

The buried surface areas between NTD and RBD and between RBD and RBD in the trimeric spike ectomains were calculated using the PISA server at the European Bioinformatics Institute (http://www.ebi.ac.uk/pdbe/prot_int/pistart.html) (51). For each trimeric spike ectodomain, a PDB file containing the coordinates from the pair of the corresponding domains was submitted to the PISA server, and the buried surface area for each pair was individually calculated.

### Calculation of angle between domains

The rotation angle between the S1 domains in SARS-CoV-2 spike structures was calculated using the angle_between_domains script in the Psico program (https://pymolwiki.org/index.php/Psico).

### Mouse immunization

Male and female BALB/c mice were intramuscularly (I.M.) immunized with each recombinant SARS-CoV-2 spike ectodomain (10 μg/mouse; 4 mice/group), or PBS buffer, in the presence of two adjuvants: aluminum hydroxide (Alum, 500 μg/mouse; InvivoGen) and monophosphoryl lipid A (MPL, 10 μg/mouse; InvivoGen). The mice were boosted once via I.M. with the same immunogen at 4 weeks. Mouse sera were collected 10 days after the 2^nd^ immunization and detected for antibody responses against the RBD and neutralizing antibodies against SARS-CoV-2 pseudovirus entry.

### ELISA

ELISA was carried out to detect the serum IgG antibodies targeting the RBD. Briefly, ELISA plates were coated with recombinant SARS-CoV-2 RBD (containing a C-terminal His tag) (1 μg/ml) at 4°C overnight, and blocked with 2% fat-free milk at 37°C for 2 h. After three washes with wash buffer (PBS + 0.1% Tween-20), the ELISA plates were incubated with each individual mouse serum at serial dilutions. After incubation at 37°C for 1 h, the ELISA plates were washed, followed by addition of a horseradish peroxidase-conjugated mouse secondary antibody (1:5,000) (Thermo Fisher Scientific). After another incubation at 37°C for 1 h, ELISA substrate (Sigma-Aldrich) was added. The ELISA reaction was stopped using 1N H_2_SO_4_, and the ELISA signal was read at the 450 nm wavelength using an ELISA plate reader (Tecan).

### Ethics statement

Mouse work was performed in strict accordance with the guidance and recommendations in the Guide for the Care and Use of Laboratory Animals (National Research Council Institute for Laboratory Animal Research). Experiments were conducted under animal use protocols approved by the Institutional Animal Care and Use Committees at the New York Blood Center.

## Supplementary Material

1
