# Supplementary material for: Molecular switches regulating the potency and immune evasiveness of SARS-CoV-2 spike protein
Source: Res Sq. 2021 Oct 1:rs.3.rs-736159. Preprint. [Version 2] doi: 10.21203/rs.3.rs-736159/v2 (PMC8491847; doi:10.21203/rs.3.rs-736159/v2)
Supplement: 1 [file NIHPPRS736159V2-supplement-1.pdf]

Supplementary figure legends

Figure S1: Cryo-EM classifications of SARS-CoV-2 spike particles. (A) Representative 2D class averages in different orientations. (B) Summary of 3D classifications of SARS-CoV-2 spike particles.

Figure S2: Cryo-EM data for two mutant SARS-CoV-2 spikes whose atomic models were built. (A) Gold-standard Fourier shell correlation (FSC) curves. The resolutions were set at 3.8 Å for FnM-deletion spike and 4.37 Å for FnM-point spike. The 0.143 cutoff value is indicated by horizontal dotted line. (B) Partial cryo-EM density maps with fitted atomic models for FnM-deletion spike (model in blue) and for FnM-point spike (model in red).

Figure S3: S1 packing in SARS-CoV-2 spike. (A) Structure of trimeric S1 in FnM-deletion spike. Three subunits are colored differently. Noted in parentheses are the monomeric subunits where each structural element is located. (B) Comparison of S1 packing in two cryo-EM structures determined in the current study (FnM-deletion spike in the closed conformation and FnM-point spike in the closed conformation) and another cryo-EM structure determined in a previous study (FnM-point spike in the closed conformation; PDB: 6VXX).

Figure S4: Role of furin motif loop in S1 packing (A) Comparison of chain traces of monomeric S1 in FnM-deletion spike (colored in blue) and that in FnM-point spike (colored in red). (B) Comparison of chain traces of monomeric S1 in FnM-deletion spike

(colored in red) and that in mouse hepatitis coronavirus (MHV) spike (colored in red; PDB: 6VSJ).

Table S1. Summary of previously determined cryo-EM structures of coronavirus spikes

Coronavirus	RBD conformation of spike protein	Reference
Human SARS-CoV-2	~50% open; ~50% closed	(Walls et al., 2020)
Bat RaTG13-CoV	Closed	(Wrobel et al., 2020)
Pangolin SARS2-like CoV	Closed	(Wrobel et al., 2021)
SARS-CoV-1	Open	(Gui et al., 2017)
MERS-CoV	Open	(Yuan et al., 2017)
NL63-CoV	Closed	(Walls et al., 2016b)
PEDV	Closed	(Wrapp and McLellan, 2019)
229E	Closed	(Li et al., 2019)
FIPV	Closed	(Yang et al., 2020)
MHV	Closed	(Walls et al., 2016a)
IBV	Closed	(Shang et al., 2018a)
PdCoV	Closed	(Shang et al., 2018b)
HKU2	Closed	(Yu et al., 2020)
SADS	Closed	(Yu et al., 2020)

References:

Gui, M., Song, W., Zhou, H., Xu, J., Chen, S., Xiang, Y., and Wang, X. (2017). Cryo-electron microscopy structures of the SARS-CoV spike glycoprotein reveal a prerequisite conformational state for receptor binding. Cell Res *27*, 119-129.

Li, Z., Tomlinson, A.C., Wong, A.H., Zhou, D., Desforges, M., Talbot, P.J., Benlekbir, S., Rubinstein, J.L., and Rini, J.M. (2019). The human coronavirus HCoV-229E S-protein structure and receptor binding. eLife *8*.

Shang, J., Zheng, Y., Yang, Y., Liu, C., Geng, Q., Luo, C., Zhang, W., and Li, F. (2018a). Cryo-EM structure of infectious bronchitis coronavirus spike protein reveals structural and functional evolution of coronavirus spike proteins. PLoS Pathog *14*, e1007009.

Shang, J., Zheng, Y., Yang, Y., Liu, C., Geng, Q., Tai, W., Du, L., Zhou, Y., Zhang, W., and Li, F. (2018b). Cryo-Electron Microscopy Structure of Porcine Deltacoronavirus Spike Protein in the Prefusion State. J Virol *92*.

Walls, A.C., Park, Y.J., Tortorici, M.A., Wall, A., McGuire, A.T., and Veesler, D. (2020). Structure, Function, and Antigenicity of the SARS-CoV-2 Spike Glycoprotein. Cell.

Walls, A.C., Tortorici, M.A., Bosch, B.J., Frenz, B., Rottier, P.J., DiMaio, F., Rey, F.A., and Veesler, D. (2016a). Cryo-electron microscopy structure of a coronavirus spike glycoprotein trimer. Nature *531*, 114-117.

Walls, A.C., Tortorici, M.A., Frenz, B., Snijder, J., Li, W., Rey, F.A., DiMaio, F., Bosch, B.J., and Veesler, D. (2016b). Glycan shield and epitope masking of a coronavirus spike protein observed by cryo-electron microscopy. Nat Struct Mol Biol *23*, 899-905.

Wrapp, D., and McLellan, J.S. (2019). The 3.1-Angstrom Cryo-electron Microscopy Structure of the Porcine Epidemic Diarrhea Virus Spike Protein in the Prefusion Conformation. J Virol *93*.

Wrobel, A.G., Benton, D.J., Xu, P., Calder, L.J., Borg, A., Roustan, C., Martin, S.R., Rosenthal, P.B., Skehel, J.J., and Gamblin, S.J. (2021). Structure and binding properties of Pangolin-CoV spike glycoprotein inform the evolution of SARS-CoV-2. Nature communications *12*, 837.

Wrobel, A.G., Benton, D.J., Xu, P., Roustan, C., Martin, S.R., Rosenthal, P.B., Skehel, J.J., and Gamblin, S.J. (2020). SARS-CoV-2 and bat RaTG13 spike glycoprotein structures inform on virus evolution and furin-cleavage effects. Nat Struct Mol Biol *27*, 763-767.

Yang, T.J., Chang, Y.C., Ko, T.P., Draczkowski, P., Chien, Y.C., Chang, Y.C., Wu, K.P., Khoo, K.H., Chang, H.W., and Hsu, S.D. (2020). Cryo-EM analysis of a feline coronavirus spike protein reveals a unique structure and camouflaging glycans. Proc Natl Acad Sci U S A *117*, 1438-1446.

Yu, J., Qiao, S., Guo, R., and Wang, X. (2020). Cryo-EM structures of HKU2 and SADS-CoV spike glycoproteins provide insights into coronavirus evolution. Nature communications *11*, 3070.

Yuan, Y., Cao, D., Zhang, Y., Ma, J., Qi, J., Wang, Q., Lu, G., Wu, Y., Yan, J., Shi, Y., *et al.* (2017). Cryo-EM structures of MERS-CoV and SARS-CoV spike glycoproteins reveal the dynamic receptor binding domains. Nature communications *8*, 15092.

Table S2. Cryo-EM data collection and model validation statistics

SARS-CoV-2 spike ectodomain (fusion motif deletion)	
**Data Collection**	
Microscope	Titan Krios
Voltage (kV)	300
Camera	K2 summit
Camera model	Super-resolution
Defocus range (μm)	-0.6~-2.6
Exposure time (s)	8
Movies	4784
Frames per movie	40
Dose rate (e^{-}/Å^2/s)	1.564
Magnified pixel size (Å)	0.521
**Reconstruction**	
Software	RELION 3.0
Symmetry	C3
Particles refined	65,774
Map Resolution (Å)	3.80
Map sharpening *B*-factor (Å^2)	-166
**Model Validation**	
MolProbity Score	1.64
All-atom clashscore	5.54
C_β deviations	0
Rotamer outliers (%)	0
Ramachandran	
Favored (%)	95.08
Allowed (%)	4.92
Outliers (%)	0.00
RMS deviations	
Bond length (Å)	0.008
Bond angles (°)	0.818

SARS2-CoV-2 spike ectodomain (fusion motif point mutations)	
**Data Collection**	
Microscope	Titan Krios
Voltage (kV)	300
Camera	K2 summit
Camera model	Super-resolution
Defocus range (μm)	-0.6~-2.6
Exposure time (s)	8
Movies	1847
Frames per movie	40
Dose rate (e^{-}/Å^2/s)	1.564
Magnified pixel size (Å)	0.521
**Reconstruction**	
Software	RELION 3.0
Symmetry	C3
Particles refined	28,863
Map Resolution (Å)	4.37
Map sharpening *B*-factor (Å^2)	-181
**Model Validation**	
MolProbity Score	1.70
All-atom clashscore	5.34
C_β deviations	0
Rotamer outliers (%)	0.47
Ramachandran	
Favored (%)	93.88
Allowed (%)	6.12
Outliers (%)	0.00
RMS deviations	
Bond length (Å)	0.008
Bond angles (°)	0.866

Figure S1

A

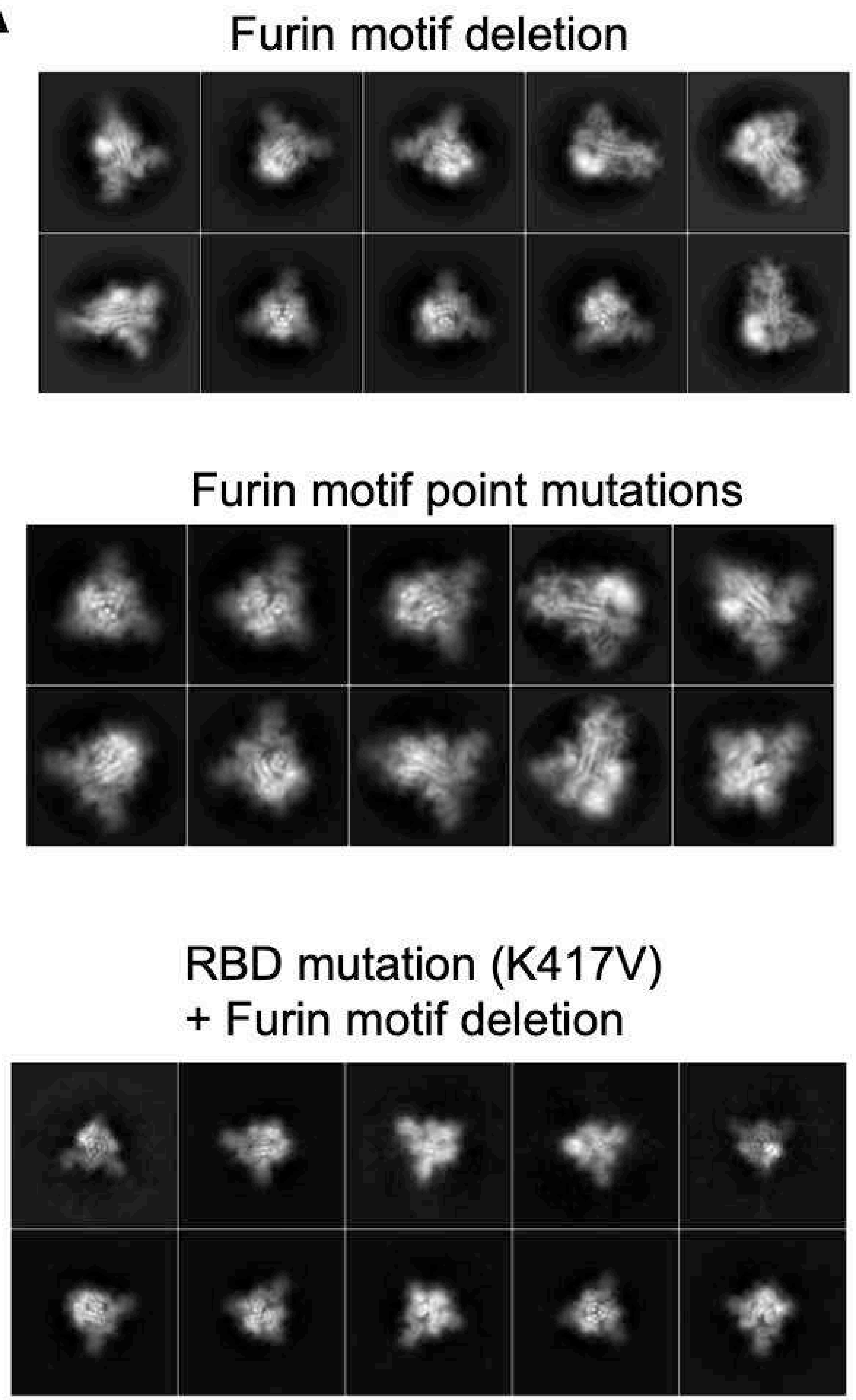

B

	3D classification	symmetry	Final number of particles	Final resolution of density
Furin motif deletion	Closed (all RBDs down)	C3	65302	3.8 Å
Furin motif point mutations	Closed (all RBDs down)	C3	23849	4.4 Å
	Open (one RBD up; two RBDs down)	C1	21894	5.3 Å
RBD mutation (K417V) + Furin motif deletion	Closed (all RBDs down)	C3	9502	4.6 Å
	Open (one RBD up; two RBDs down)	C1	101413	4.6 Å

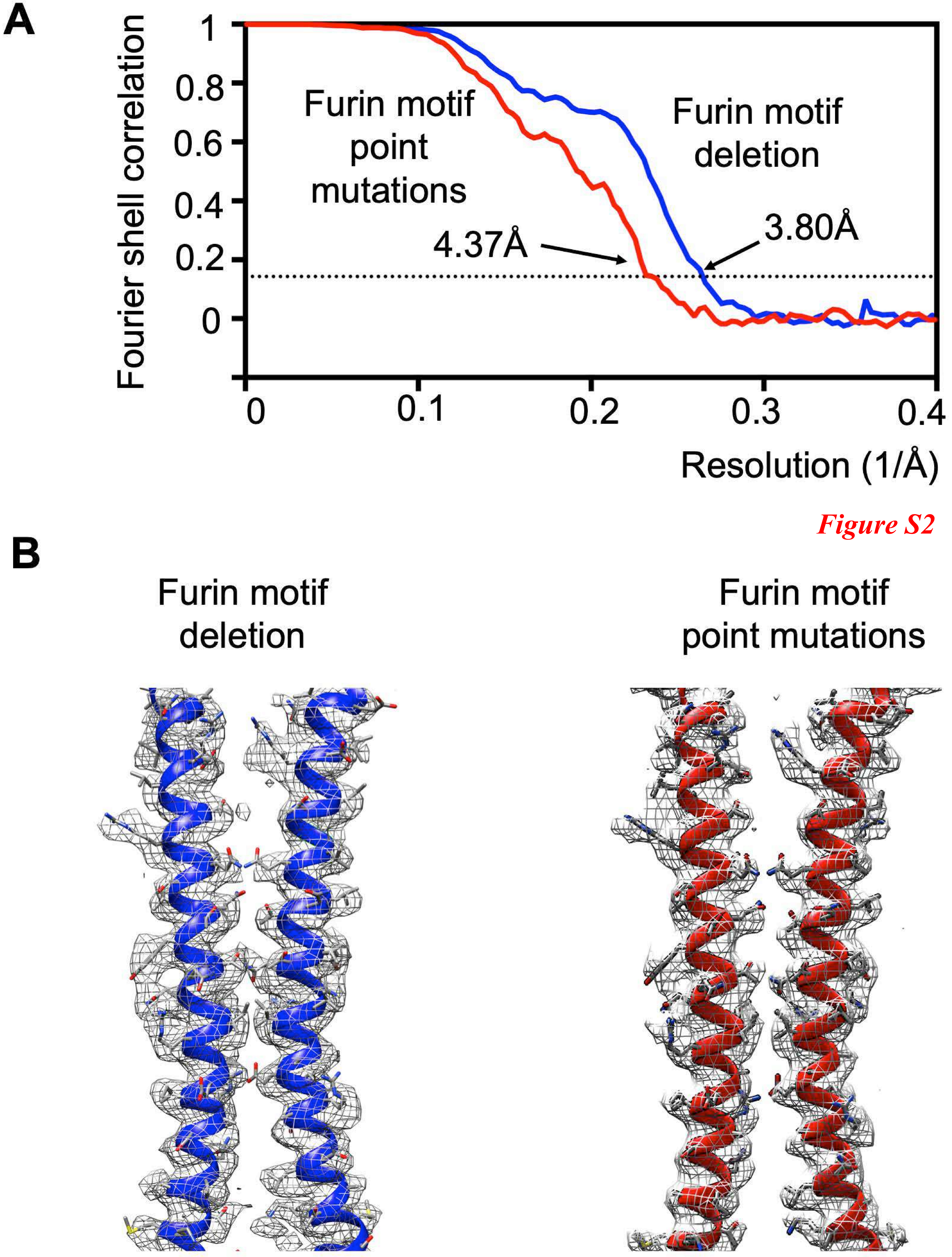
A
Fourier shell correlation
1
0.8
0.6
0.4
0.2
0
0
0.1
0.2
0.3
0.4
Resolution (1/Å)
Furin motif point mutations
Furin motif deletion
4.37Å
3.80Å
B
Furin motif deletion
Furin motif point mutations

Figure S2

Figure S3

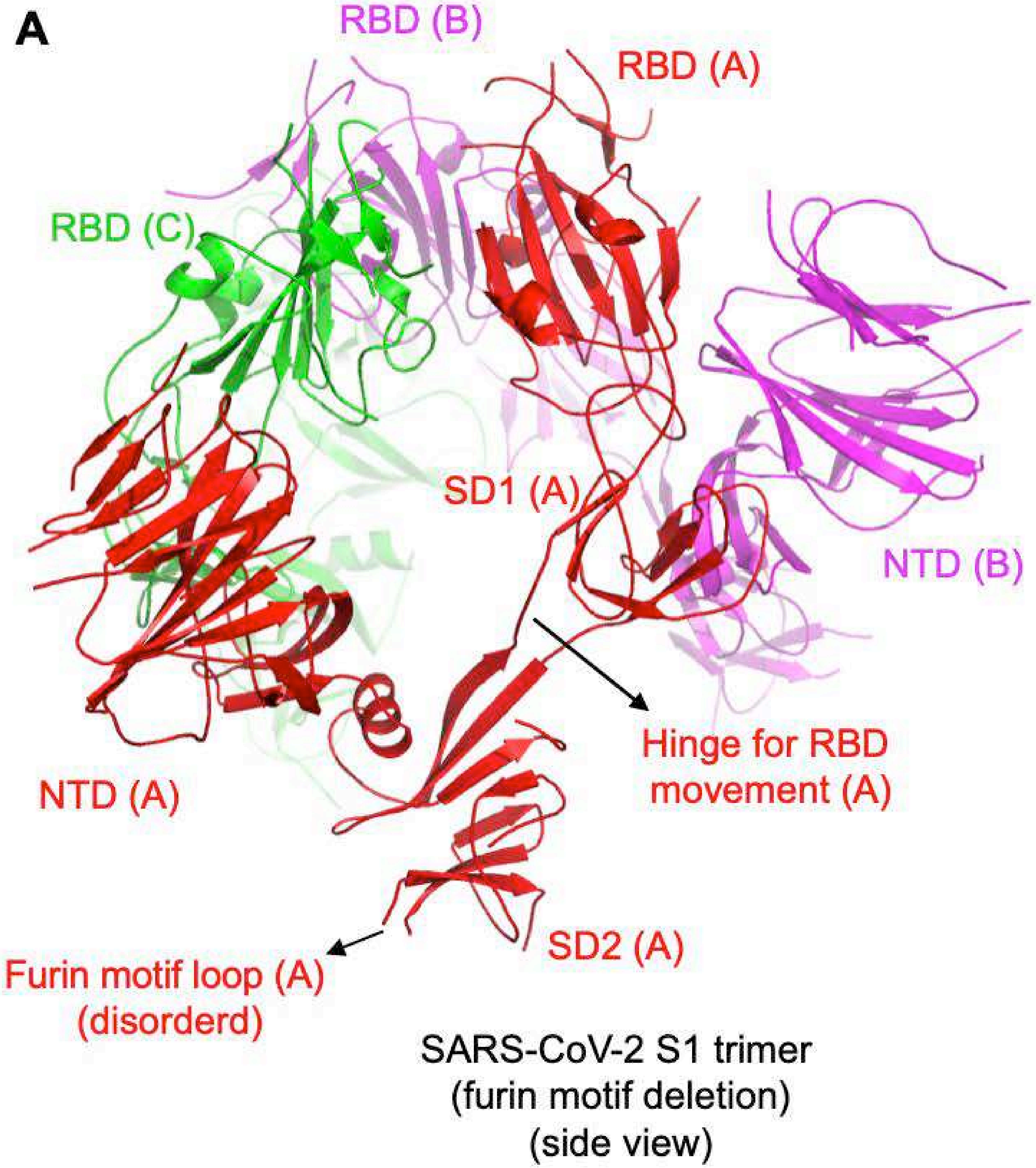

SARS-CoV-2 S1 trimer
(furin motif deletion)
(side view)

B

SARS-CoV-2 Spike	Distribution of particles	S1 packing in closed spike structure		
		NTD/RBD interface	RBD/RBD interface	Total interface in trimeric S1
Furin motif deletion (current study)	Closed: 100%	806 Å^2	234 Å^2	3120 Å^2
Furin motif point mutations (current study)	Open: 52%<br>Closed: 48%	611 Å^2	173 Å^2	2352 Å^2
Furin motif point mutations (6VXX)	Open: 50%<br>Closed: 50%	689 Å^2	138 Å^2	2481 Å^2

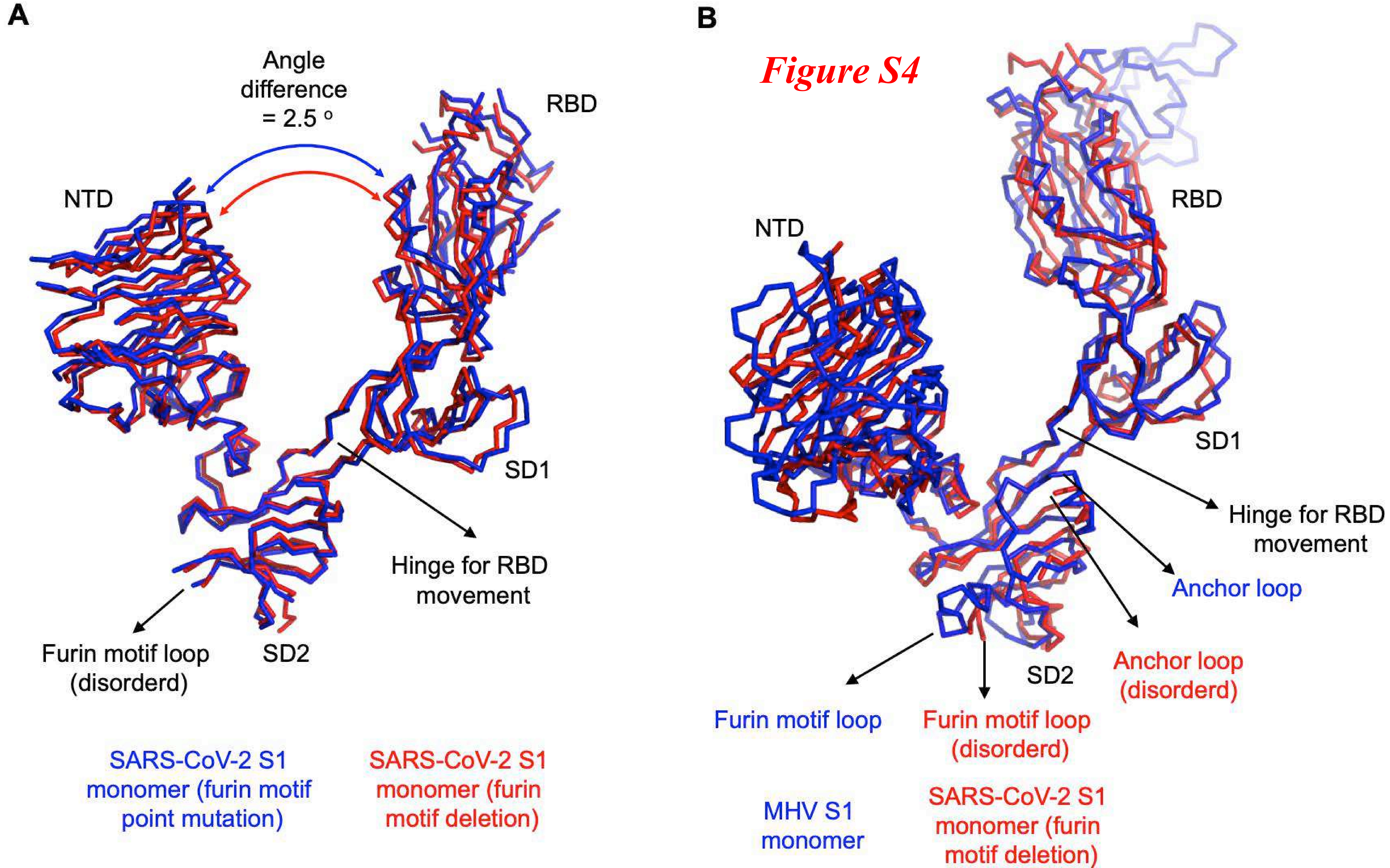
A
Angle difference = 2.5 °
RBD
NTD
SD1
Hinge for RBD movement
Furin motif loop (disorderd)
SD2
SARS-CoV-2 S1 monomer (furin motif point mutation)
SARS-CoV-2 S1 monomer (furin motif deletion)
B
NTD
RBD
SD1
Hinge for RBD movement
Anchor loop
Anchor loop (disorderd)
SD2
Furin motif loop
Furin motif loop (disorderd)
MHV S1 monomer
SARS-CoV-2 S1 monomer (furin motif deletion)

Figure S4
